# A Case of Carcinosarcoma of the Peritoneum With Serous Tubal Intraepithelial Carcinoma

**DOI:** 10.1155/2024/1907965

**Published:** 2024-10-22

**Authors:** Akiko Kanemura, Tohru Morisada, Mai Momomura, Fumio Asano, Hiromi Shibuya, Hironori Matsumoto, Kiyotaka Nagahama, Junji Shibahara, Yoichi Kobayashi

**Affiliations:** ^1^Department of Obstetrics and Gynecology, Faculty of Medicine, Kyorin University, Tokyo, Japan; ^2^Department of Pathology, Faculty of Medicine, Kyorin University, Tokyo, Japan

**Keywords:** carcinosarcoma, fallopian tube fimbriae, peritoneal carcinosarcoma, serous carcinoma, serous intraepithelial carcinoma

## Abstract

In this case, a 66-year-old female patient presented with the chief complaint of abdominal distention. Computed tomography and magnetic resonance imaging revealed no enlarged ovaries or obvious primary lesions; however, massive ascites and peritoneal disseminated nodules were observed. Ascites cytology revealed adenocarcinoma and immunohistochemical findings suggested serous carcinoma. The patient then underwent tumor reduction surgery after preoperative chemotherapy for suspected Stage IIIC primary peritoneal carcinoma. Postoperative histopathology revealed carcinoma consisting mainly of high-grade serous carcinoma (HGSC) and sarcoma. In addition, serous intraepithelial carcinoma (STIC) of the fallopian tube was observed in the fimbriae of the left fallopian tube. Recently, it has been noted in the literature that most cases of peritoneal carcinoma are metastases or dissemination of carcinoma originating from the fimbriae of the fallopian tube. This is a rare case of peritoneal carcinosarcoma with STIC, and its report leads to a better understanding of the disease's features and possible therapeutic approaches.

## 1. Introduction

Peritoneal carcinosarcoma is extremely rare, with only approximately 30 cases having been reported in the literature thus far [1], the majority being in women over the age of 40 [2]. The prognosis of this condition is extremely poor [3], and effective treatments have not yet been established. Primary peritoneal carcinoma has been recognized as a tumor that arises multicentrally from mesothelial cells lining the omentum, diaphragm, and mesentery, as well as from the ovarian surface epithelial cells that are contiguous with these cells. In what are conventionally considered primary peritoneal carcinomas, most of the histologic types are high-grade serous carcinomas (HGSC). In recent years, it has been suggested that many primary peritoneal carcinomas may be primary tumors of the fallopian tubes [4, 5]. Here, we report a case of peritoneal carcinosarcoma with serous tubal intraepithelial carcinoma (STIC) of the fallopian tube which was found after laparotomy, although preoperative chemotherapy underwent for peritoneal carcinosarcoma presenting primarily as peritoneal lesions.

## 2. Case Presentation

The patient was a 66-year-old woman, gravida four, para three. She had entered menopause at 52 years of age, had a history of bronchial asthma, and had a family history of breast cancer (mother and sister) and gastric cancer (father). The patient had visited her previous doctor owing to a weight gain of 6 kg in 1 month along with abdominal distension. The patient was referred to our hospital for further examination because of the presence of a large amount of ascitic fluid and peritoneal dissemination. Owing to her poor general condition, the patient was hospitalized and underwent a thorough examination that included ascites puncture cytology. Gynecological examination revealed that the uterus was atrophic, and the bilateral adnexa were unpalpable. Additionally, transvaginal ultrasonography revealed numerous disseminated nodules in the pouch of Douglas. No evident ovarian enlargement was observed.

**Figure 1:**
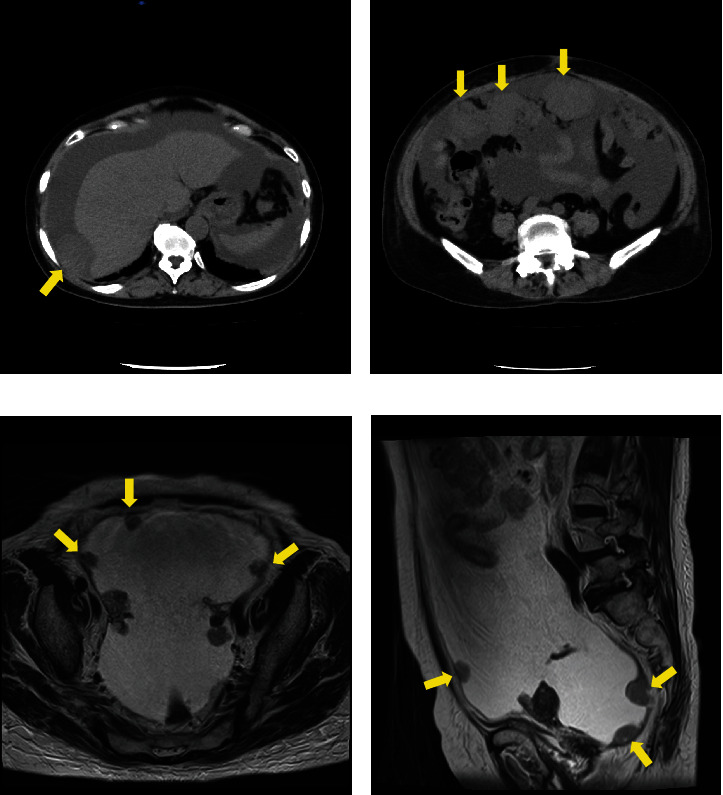
(a, b) A large amount of ascites fluid and disseminated nodules in the upper and lower abdomen were observed (arrow). There was no evidence of a primary tumor in any intra-abdominal organ. (c, d) Numerous peritoneal disseminated nodules were seen in the pelvis (arrow). Bilateral ovaries were of normal size.

Laboratory analysis showed evidence of hypoalbuminemia with Alb 1.8 g/dL. The cancer antigen-125 (CA-125) level of the patient was elevated to 929.4 U/mL, CA19-9 was < 2.0 U/mL, and carcinoembryonic antigen was 1.6 U/mL. Computed tomography (CT) showed no evidence of a primary tumor in any intra-abdominal organs; however, ascites and peritoneal dissemination were observed (Figures 1(a) and 1(b)). Contrast–enhanced magnetic resonance imaging also revealed numerous disseminated nodules in the pelvis, but both ovaries were normal in size (Figures 1(c) and 1(d)).

The ascitic fluid cytology result was Class V, suggesting an adenocarcinoma. Immunostaining revealed weak positivity for CK7 and CK20 in the atypical cells in the specimen. Some atypical cells were positive for WT-1, PAX8, CA125, p53, PgR, and CD56. The ascites cytology results were indicative of a serous carcinoma.

As the patient's European performance status was three, she was treated with six cycles of preoperative chemotherapy with carboplatin, paclitaxel, and bevacizumab for suspected Stage IIIC primary peritoneal cancer. At the end of chemotherapy, the CA125 levels decreased to within the normal range. Subsequently, the patient underwent laparotomy including total hysterectomy, bilateral adnexectomy, omentectomy, peritonectomy, and dissemination resection.

**Figure 2:**
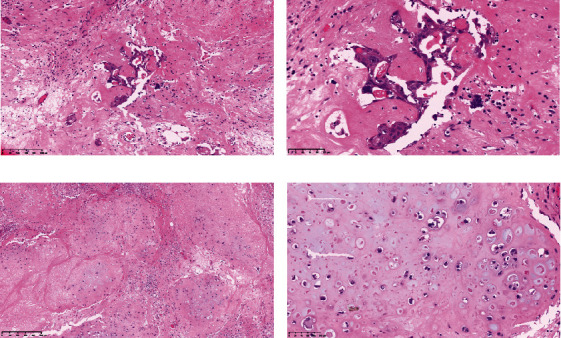
(a, b) Histology of the greater omentum, peritoneum, and left ovary showing carcinosarcoma, with high-grade serous carcinoma component and (c, d) a chondrosarcoma component.

**Figure 3:**
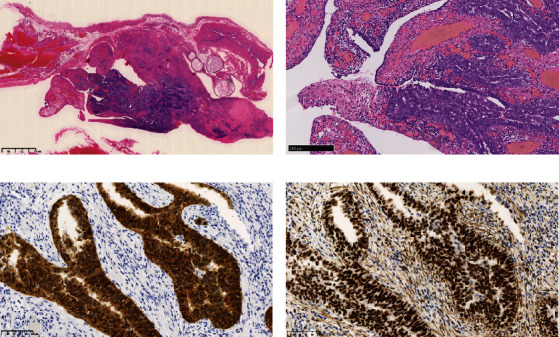
Histology of the left fallopian tube fimbriae showing (a, b) serous tubal intraepithelial carcinoma positive for (c) p53 and (d) WT-1.

**Figure 4:**
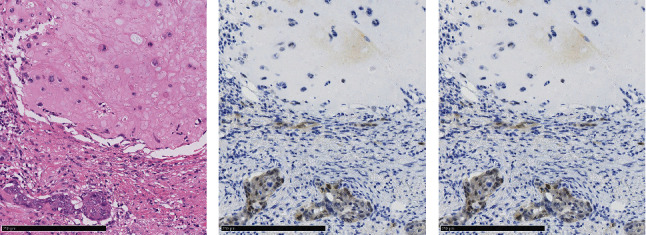
(a) Histology of the peritoneal nodule showing carcinosarcoma with high-grade serous carcinoma component and a chondrosarcoma component, having weak positivity for (b) p53 and (c) WT-1.

Operative findings revealed that the uterus and ovaries were atrophic, and numerous disseminations were observed in the vesicouterine fossa, surfaces of the small intestine, mesentery, and rectum. A 4–5-cm dissemination was also observed in the omentum. Surgical intervention was determined as optimal. Histopathological examination revealed a carcinosarcoma involving the greater omentum, retroperitoneum, and surface of the left ovary. There were no lesions in the ovarian parenchyma. The tumor comprised HGSC and chondrosarcoma, and the left fallopian tube contained STIC confirmed by positive staining of p53 and WT-1 on immunohistochemistry (Figures 2 and 3), whereas the peritoneal nodule shows carcinosarcoma with a high-grade serous carcinoma component and a chondrosarcoma component, having weak positivity for p53 and WT-1 (Figure 4). The postoperative CA125 value was 16.1 U/mL. After the postoperative diagnosis of Stage IIIC fallopian tube cancer was made, the patient underwent three cycles of chemotherapy with carboplatin, paclitaxel, and bevacizumab and 21 cycles of maintenance therapy with bevacizumab. One year and 9 months postoperatively, 18F-fluorodeoxyglucose (FDG) positron emission tomography (PET)–CT showed metastasis in the S7 region of the liver and multiple lymph nodes, and the patient then underwent another six cycles of chemotherapy with carboplatin and paclitaxel for platinum-sensitive recurrence. After confirming partial response, maintenance therapy with olaparib was administered. Five months after starting maintenance therapy, the disease progressed, and chemotherapy with liposomal doxorubicin and bevacizumab was administered for platinum-resistant recurrence.

## 3. Discussion

Primary peritoneal carcinoma arises multicentrally from mesothelial cells lining the abdominal peritoneum, omentum, diaphragm, and mesentery, as well as from ovarian surface epithelial cells. This type of carcinoma shows the same pathogenesis as ovarian superficial epithelial malignancies, and most histological types are serous carcinomas, whereas clear cell, mucinous, and endometrioid carcinomas occur rarely. It has now been suggested that peritoneal and ovarian carcinomas originate from the intraepithelial carcinoma of the distal end of the fallopian tube fimbriae.

In a condition such as this case, a laparoscopic surgery to identify the primary organ would be useful [6]. However, due to poor general condition, the patient was treated for primary peritoneal carcinoma; however, postoperative pathological findings revealed a peritoneal carcinosarcoma with STIC in the fimbriae of the left fallopian tube.

Carcinosarcoma, also known as malignant Müllerian duct (mesoderm) mixed tumor, comprises both carcinomatous and sarcomatous components. Most gynecological carcinosarcomas arise in the uterus, and extragenital carcinosarcomas such as primary peritoneal carcinosarcomas are rare [7, 8]. While uterine carcinosarcomas account for 2%–3% of uterine malignancies, only approximately 30–40 cases of primary peritoneal carcinosarcoma have been reported in the literature [9]. Until recently, carcinosarcoma histogenesis was explained by the collision, combination, conversion, composition, and hypothetical theories. Generally, uterine carcinosarcoma is derived from a single cell, and the combination theory wherein both the carcinoma and sarcoma components differentiate during tumorigenesis is supported.

The origin of peritoneal carcinosarcoma remains unknown; however, there were several reported cases associated with endometriosis, including primary peritoneal Mullerian adenosarcoma with sarcomatous overgrowth associated with endometriosis [10], vaginal adenosarcoma arising in extrauterine endometriosis [11], endometrioid adenosarcoma arising from vesical endometriosis [12], and Mullerian adenosarcoma arising in perirectal endometriosis [13]. The frequency of the fallopian tubes as a site of extrauterine endometriosis is also noteworthy [14]. Furthermore, it is possible that peritoneal carcinoma may have converted to carcinosarcoma, as in the uterine carcinosarcoma described above. The prognosis of peritoneal carcinosarcoma is extremely poor with a median postoperative survival of 14 months, and most patients die within a year [15]. Although no effective treatment has been established, treatment with carboplatin + paclitaxel and ifosfamide + cisplatin, among other therapies, is considered for uterine and ovarian carcinosarcomas [16]. Favorable response of PARP inhibitors in advanced or recurrent ovarian carcinosarcoma has also been reported [17, 18]. Reportedly, multimodality treatment, including postoperative radiation therapy, has led to disease-free survival for 5 years [1].

Although precancerous lesions of ovarian HGSC and primary peritoneal HGSC were thought to be located in the ovary or peritoneum, STIC has been proposed to originate from the distal end of the fimbriae of the fallopian tube [19]. Recently, criteria for determining the primary site of HGSC have been proposed. The diagnosis of primary peritoneal HGSC was recently being made only when detailed histological examination of the fallopian tubes and ovaries revealed no lesions in these areas. Most cases with STIC are judged to be primary fallopian tube lesions [20].

The etiologic relationship between STIC and peritoneal carcinosarcoma in this case has several possibilities. For example, it is possible that the primary peritoneal carcinosarcoma and STIC of the fallopian tubes occurred simultaneously. Alternatively, the STIC could have implanted in the peritoneum from the fallopian tube and converted to a carcinosarcoma. In the present case, the areas of dissemination outside of the fallopian tube stained weakly positive for p53 and WT-1, a different staining pattern from that seen in the HGSC of the fallopian tube. It has been reported that when STIC and carcinosarcoma were from the same clone, the immunoreactivity of p53 was also the same [21, 22]. This suggests that a primary peritoneal carcinosarcoma may have existed apart from HGSC in the fallopian tubes as simultaneous double cancer in this case.

## 4. Conclusion

Herein, we report a case of carcinosarcoma with STIC that had been preoperatively assumed to be primary peritoneal carcinoma, which is extremely rare. Histopathological examination then revealed STIC in the left fallopian tube. Although an effective treatment for peritoneal carcinosarcoma has yet to be established, a combination of tumor resection and chemotherapy may be effective. Further accumulation of cases is required to establish more effective treatments.

## Data Availability

The data that support the findings of this study are available on request from the corresponding author. The data are not publicly available due to privacy or ethical restrictions.
